# ggplotAgent: a self-debugging multi-modal agent for robust and reproducible scientific visualization

**DOI:** 10.1093/bioadv/vbaf332

**Published:** 2026-01-02

**Authors:** Zelin Wang, Yuanyuan Yin, Jien Wang, Haiyan Yan, Xuan Xie, Yiqing Zheng

**Affiliations:** Guangdong Provincial Key Laboratory of Cancer Pathogenesis and Precision Diagnosis and Treatment, Joint Big Data Laboratory, Department of Medical Oncology, Shenshan Medical Center, Memorial Hospital of Sun Yat-sen University, Shanwei, 516600, China; Guangdong Provincial Key Laboratory of Cancer Pathogenesis and Precision Diagnosis and Treatment, Joint Big Data Laboratory, Department of Medical Oncology, Shenshan Medical Center, Memorial Hospital of Sun Yat-sen University, Shanwei, 516600, China; Medical Research Center, Sun Yat-sen Memorial Hospital, Sun Yat-sen University, Guangzhou, 510120, China; Guangdong Provincial Key Laboratory of Cancer Pathogenesis and Precision Diagnosis and Treatment, Joint Big Data Laboratory, Department of Medical Oncology, Shenshan Medical Center, Memorial Hospital of Sun Yat-sen University, Shanwei, 516600, China; Guangdong Provincial Key Laboratory of Cancer Pathogenesis and Precision Diagnosis and Treatment, Joint Big Data Laboratory, Department of Medical Oncology, Shenshan Medical Center, Memorial Hospital of Sun Yat-sen University, Shanwei, 516600, China; Department of Thoracic Surgery, Sun Yat-Sen Memorial Hospital, Sun Yat-Sen University, Guangzhou, 510120, China; Guangdong Provincial Key Laboratory of Cancer Pathogenesis and Precision Diagnosis and Treatment, Joint Big Data Laboratory, Department of Medical Oncology, Shenshan Medical Center, Memorial Hospital of Sun Yat-sen University, Shanwei, 516600, China

## Abstract

**Motivation:**

Creating publication-quality visualizations is essential for bioinformatics but remains a bottleneck for researchers with limited coding expertise. While Large Language Models (LLMs) are proficient at generating code, they often fail in practice due to library dependencies, dataset mismatches, or syntax errors. These issues require manual intervention, slowing data interpretation.

**Results:**

We present ggplotAgent, a novel multi-modal, self-debugging artificial intelligence agent that automates publication-ready ggplot2 visualizations. It features a dual-layered framework that resolves code execution errors and uses a vision-enabled agent to verify aesthetic correctness. In benchmarks against the DeepSeek-V3 model, ggplotAgent achieved a 100% code executability rate(versus 85%) and a “Publication-Ready” score of 1.9 (versus 0.7). Surprisingly, it showcased the ability to act as an expert collaborator by intelligently enhancing plots beyond the user’s literal prompt, achieving a positive Insight Score of +0.3 over than the baseline (−0.05). These results demonstrate its ability to reliably produce accurate, high-quality visualizations directly from natural language.

**Availability and implementation:**

ggplotAgent is freely accessible as a public web application at https://ggplotagent.databio1.com/ and an offline Streamlit app. The source code is available on GitHub at https://github.com/charlin90/ggplotAgent. This software is distributed under the MIT License.

## 1 Introduction

The advance of high-throughput omics technologies has led to an unprecedented volume of complex biological data (Goodwin *et al.* 2016, Burgess 2019). Effective data visualization is not merely an accessory but a critical tool for hypothesis generation, pattern discovery, and the communication of scientific findings. Libraries such as ggplot2 in R have become the gold standard for creating sophisticated, publication-quality graphics due to their power and adherence to the “grammar of graphics” (Wickham 2016). However, mastering these libraries requires a significant investment in learning programming, creating a barrier for biologists and clinicians.

To bridge this gap, several tools have emerged. Web-based platforms like GraphBio (Zhao and Wang 2022), ImageGP (Chen *et al.* 2022), ggVolcanoR (Mullan *et al.* 2021), Hiplot (Li *et al.* 2022), OmicStudio (Lyu *et al.* 2023), and GUI-based software like GraphPad Prism offer user-friendly interfaces but are often constrained by predefined plot types and limited customization options. This rigidity can stifle exploratory analysis and hinder the creation of novel or highly specific visualizations required for publication. Furthermore, workflows in these tools are often difficult to document, posing a challenge to reproducibility.

The recent advancement of Large Language Models (LLMs) presents a transformative opportunity to overcome these challenges (Wang *et al.* 2023). However, directly applying general-purpose LLMs like ChatGPT for scientific visualization reveals some critical problems. First, the generated code, while often syntactically plausible, is technically fragile. It frequently fails in a researcher’s environment due to missing library dependencies, mismatches with specific data formats (e.g. column names in bioinformatics datasets), or subtle syntactic hallucinations. Second, these models act as literal interpreters, translating a user’s prompt into a basic plot. They lack the domain expertise of a seasoned bioinformatician to infer intent, apply visualization best practices, and transform a simple request into an insightful, publication-ready figure. These challenges necessitate a more robust, specialized solution.

Here, we introduce ggplotAgent, a self-debugging multi-modal agent that automates the generation of publication-ready ggplot2 plots. It operates on a core mechanism we term the dual-layered debugging framework. The first layer directly targets code errors: an agent iteratively executes the generated R script in a safe environment, automatically identifying and correcting syntactic bugs, missing library dependencies, and other runtime failures until the code is fully executable. Subsequently, the second layer addresses plot quality: a vision-enabled agent visually inspects the resulting image, intelligently correcting aesthetic flaws, layout issues, and misinterpretations of the user’s intent. This two-stage, self-correction process ensures that the final output is not only technically sound but also semantically and visually aligned with the standards of a publication-ready figure, effectively bridging the gap between a simple prompt and an expert-level visualization. Overall, ggplotAgent empowers researchers to produce publication-ready visualizations without writing a single line of code.

## 2 Methods

**Figure 1.**
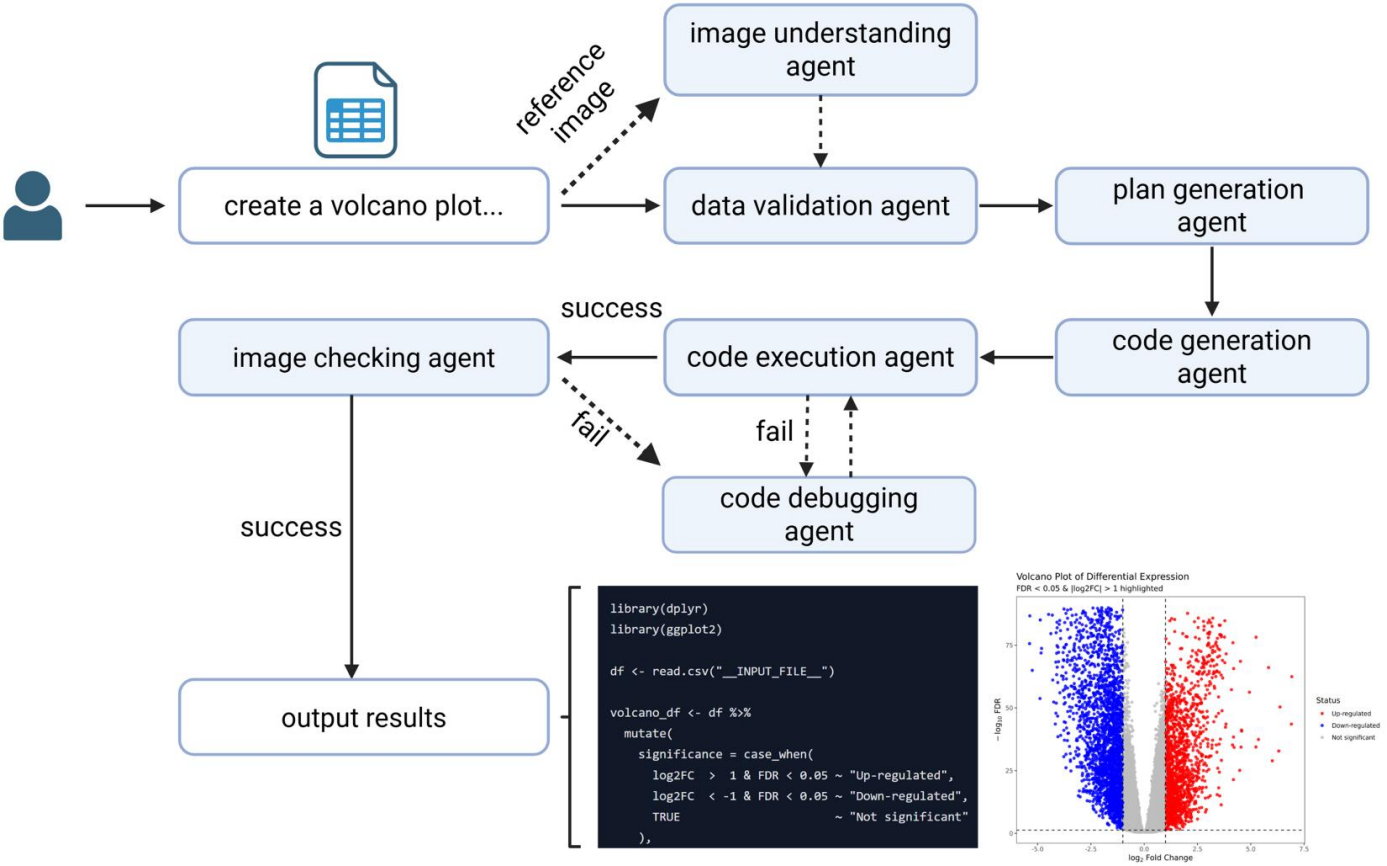
Overview of the ggplotAgent workflow. The system begins by processing a user’s prompt, a required CSV file, and an optional reference image. Once the data validation agent deems the request reasonable, the plan generation agent creates a detailed plan. Upon plan generation, the code generation agent generates an R script. This script enters an iterative self-debugging loop: the code execution agent runs the script, and the code debugging agent checks for syntax errors. If successful, an image checking agent visually validates the output plot against the user request. Any syntax or visual errors are fed back to the code debugging agent, which generates a corrected script for the next iteration. The loop concludes when a plot passes both checks, yielding a reproducible R script and a publication-quality figure.

ggplotAgent is a multi-agent system orchestrated in Python, made available to users through an intuitive web interface and an offline Streamlit app. It guides the user from a high-level request to a final, validated plot and its underlying R code. The system’s text generation and reasoning capabilities are powered by the deepseek-v3-250324 language model, while image analysis is handled by the doubao-1–5-thinking-vision-pro-250428 vision model. This system is composed of several specialized agents operating in a sequence that includes a critical feedback loop (Fig. 1).

### 2.1 Multi-modal input and planning

The process begins with user input, which can be a text prompt (e.g. “Create a volcano plot from my data, highlighting significant genes”) and an optional reference image.

Image understanding agent: If a reference image is provided, this agent analyzes its visual characteristics—such as plot type, color schemes, themes, and annotations—and fuses this stylistic information with the user’s text prompt to create a single, detailed set of instructions.

Data validation agent: This agent semantically validates that the provided data can fulfill the user’s plotting request. It intelligently recognizes common bioinformatics column aliases (e.g. logFC, FDR) and necessary data transformations. This ensures data-request compatibility.

Plan generation agent: Using the detailed instructions, this agent creates a comprehensive, two-part plan. The first part outlines necessary data preprocessing steps (e.g. calculating −log10 *P*-values, creating a significance column). The second part details the ggplot2 visualization strategy, specifying the geoms, aesthetic mappings (*x*, *y*, color), labels, and theme.

### 2.2 Iterative self-debugging loop

Upon generation of the internal plan, ggplotAgent enters its core iterative loop designed to ensure robustness and correctness.

Code generation agent: This agent translates the plan into a complete, self-contained R script. The script includes all necessary library calls [library(tidyverse), library(ggrepel)], data loading, preprocessing, plotting, and a ggsave() command to export the final figure.

Code execution agent: This agent runs the generated R script in a secure and reproducible Docker container based on the rocker/tidyverse image. It captures all output and error streams from the execution. This provides either the successfully generated plot or precise diagnostics for an automated debugging cycle.

Code debugging agent: This agent, equipped with the original plan, the faulty script, and the error message, analyzes the failure and generates a corrected version of the entire script. This cycle repeats up to a predefined number of retries.

Image checking agent: This agent compares the generated plot image against the user request. If it detects a visual or logical discrepancy (e.g. axes are swapped, colors do not represent the correct variable, a requested title is missing), it generates a feedback report. This feedback is passed to the Code Debugging Agent, which then revises the script’s logic to correct the aesthetic or semantic error. The script is then re-executed and re-validated.

This dual-validation loop terminates upon successful visual validation or if the maximum retry limit is reached. For a more detailed description, please refer to the Supplementary Methods.

## 3 Results

The workflow of ggplotAgent is illustrated in Fig. 1. The process begins when a user provides a dataset and a prompt in natural language, such as “create a volcano plot…”. The data validation agent first inspects the input data for integrity and compatibility, ensuring necessary columns are present. Concurrently, if a reference image is provided, the image understanding agent analyzes its visual characteristics, such as color schemes, layouts, and font styles, to extract a set of aesthetic guidelines. These inputs are then passed to the plan generation agent, which deconstructs the user’s request into a structured, step-by-step plotting strategy. This plan is fed to the code generation agent, which writes the corresponding ggplot2 code in R. A key feature of our system is the self- debugging loop involving the code execution agent and the code debugging agent as well as image checking agent. The generated code is executed and checked for syntax errors. If it runs successfully, the resulting plot is then validated by the image checking agent against the request’s specifications. If it fails at any of these stages—either due to a code error or a visual discrepancy—the code debugging agent analyzes the issue (using the error message or the visual feedback) and generates a corrected script, which is then resubmitted for a new execution cycle. This iterative process continues until the code executes flawlessly and the generated plot passes the visual verification. Only when both conditions are met are the final code and the publication-quality image presented as the output results.

To quantitatively evaluate our approach, we compared ggplotAgent against a strong baseline, DeepSeek-V3, on a benchmark of 20 diverse visualization tasks. Performance was assessed across four metrics: Code Executability, Success Score (task completion), Insight Score (intelligent enhancements), and Publication-Ready Score (aesthetic quality) (Supplementary Methods). The results show that ggplotAgent demonstrates superior performance across all dimensions (Table 1). It achieved a perfect 100% executability rate and a near-perfect Success Score of 1.95, significantly outperforming the baseline. Crucially, ggplotAgent earned a positive Insight Score (0.3), indicating its ability to provide valuable, unprompted enhancements. A key example was its autonomous application of a logarithmic scale to skewed data, revealing trends that were otherwise obscured (Fig 1A and B, available as supplementary data at *Bioinformatics Advances* online). In contrast, DeepSeek-V3 scored slightly negative (−0.05) on this metric. Finally, the stark difference in the Publication-Ready Score (1.9 versus 0.7) highlights our agent’s ability to produce polished, professional charts ready for immediate use. These results validate the effectiveness of our specialized approach. In addition, we also tested the system with alternative models (Qwen3-Max and Qwen3-VL-Plus) for a subset of tasks. The results were placed on https://github.com/charlin90/ggplotAgent/tree/main/qwen3_outputs. We demonstrated that the framework is robust for different models, although performance varies.

**Table 1.** Benchmark comparison of ggplotAgent and baseline LLM.^a^

Model	Code executability rate (%)	Avg. success score	Avg. insight score	Publication-ready score
ggplotAgent (Ours)	100.00%	1.95	0.3	1.9
DeepSeek-V3	85.00%	1.5	−0.05	0.7

a Detailed evaluation scores for each plot are available at https://github.com/charlin90/ggplotAgent/tree/main/benchmark.

We demonstrate the capabilities of ggplotAgent with a common bioinformatics use case: visualizing differential gene expression data. We used a sample CSV file (volcano_example.csv) containing gene, log2FC, and FDR columns. We first prompted ggplotAgent with a detailed text-based request: “Generate a publication-quality volcano plot with the *x*-axis representing log2 Fold Change and the *y*-axis representing −log10 FDR. Color points red for significantly upregulated features (log2FC > 1 and FDR < 0.05), blue for significantly downregulated features (log2FC < −1 and FDR < 0.05), and grey for all others. Add a horizontal dashed line indicating the significance threshold at FDR = 0.05, and vertical dashed lines at log2FC = 1 and −1. Ensure the plot has a clean white background with no grid lines.” Following the described workflow, the system successfully interpreted these complex instructions and generated the requested volcano plot (Fig 1C, available as supplementary data at *Bioinformatics Advances* online).

Furthermore, a powerful feature of ggplotAgent is its ability to create plots based on a reference image, simplifying user prompts. Using the same dataset, we provided a simpler prompt: “Create a publication-quality plot similar to the reference image. Use thresholds log2FC = 1 and FDR = 0.05. Only label the top 3 most up-regulated and down-regulated genes, respectively. Show the numbers of up- and down-regulated genes in title. Text labels are black. Legend is on right”. In this scenario, the image understanding agent extracted the stylistic elements from the example, which combined with the simplified text instructions, guided the generation process. The resulting plot, which effectively mimics the style of the reference while applying the new labeling rules, was presented in Fig 1D and E, available as supplementary data at *Bioinformatics Advances* online. This dual-modality input system empowers users to achieve desired styles with either precise textual commands or visual examples, lowering the barrier to creating sophisticated, publication-quality visualizations and allowing researchers to focus on scientific interpretation rather than coding intricacies. More examples were provided in https://github.com/charlin90/ggplotAgent.

## 4 Discussion

### 4.1 Conclusion

ggplotAgent represents a significant step toward democratizing high-quality scientific visualization. By providing an intuitive, multi-modal interface and a novel self-debugging workflow, it empowers researchers to translate complex ideas into reproducible, publication-quality figures without needing to be expert programmers. The core innovation, a dual-layered debugging framework, addresses the critical limitations of general-purpose LLMs: it not only resolves technical code failures but also ensures the final output is semantically and visually aligned with the user’s intent. This moves beyond simple code generation to offer a solution that acts as an automated visualization expert. By empowering researchers to create sophisticated and reproducible plots without extensive programming knowledge, ggplotAgent lowers the barrier to entry for advanced data analysis.

### 4.2 Limitations

While ggplotAgent demonstrates significant promise, we acknowledge several limitations that warrant consideration. Firstly, the current implementation relies on API calls to external, proprietary large language models. This introduces challenges related to runtime latency, as complex plots may require multiple debugging iterations, and computational cost. Furthermore, the system’s performance is inherently tied to the capabilities and potential biases of these underlying models; any changes or degradation in their performance could directly impact ggplotAgent’s robustness. Secondly, while the vision-enabled agent is effective at capturing high-level styles (e.g. general layout), achieving pixel-perfect replication of every intricate detail from a reference image remains a significant challenge. Nuances like precise font sizing, specific annotation placements, and complex legend structures can sometimes be approximated rather than perfectly duplicated. Thirdly, the public web server is currently deployed on a single instance with limited computational resources. This setup is adequate for demonstration purposes and moderate use, but it does not scale well to handle a high volume of concurrent user requests or large data file (e.g. single-cell RNA-seq matrices). Under heavy load, users may experience significant latency or request timeouts. This limitation was a key motivation for developing the offline application, which offloads the computational work to the user’s local machine. Lastly, the current sandboxed environment for ggplotAgent is intentionally constrained to a core set of R packages, primarily the tidyverse and ggrepel. While the broader ggplot2 ecosystem contains hundreds of extension packages (e.g. ggvenn, ggridges), they are not pre-installed in the execution environment. This was a deliberate design choice to rigorously test the robustness of our core framework on a consistent and controlled set of functionalities. Although our preliminary tests showed the debugging agent could autonomously identify and attempt to install missing packages, we restricted the environment to ensure that observed performance gains were due to our framework’s logic, not simply automated package installation.

### 4.3 Future work

Building on the current framework, our future research will pursue several key directions to enhance ggplotAgent’s capabilities and broaden its impact. A primary focus will be to improve the visual acuity of the style-transfer process. We plan to explore advanced prompt engineering techniques and consider fine-tuning the vision-language model on a curated dataset of scientific figures, aiming for more granular control over aesthetic details like font matching and precise annotation placement. To support a wider range of bioinformatics visualizations, we will also expand the sandboxed execution environment to include a broader array of pre-installed ggplot2 extension packages, enabling the generation of more diverse and specialized plots. We also plan to explore a more robust deployment architecture, such as using containerization (e.g. Docker) and orchestration tools (e.g. Kubernetes) to manage multiple worker instances. Implementing a job queue system (e.g. Celery with Redis) would also allow for better management of asynchronous tasks, improving responsiveness and enabling the service to handle a larger number of concurrent users gracefully. Looking further ahead, we aim to transcend current input modalities by exploring more creative and intuitive forms of interaction, such as the ability to interpret user-provided sketches or hand-drawn diagrams. This would represent a significant leap toward a truly intuitive “idea-to-figure” workflow, further democratizing complex data visualization for all researchers.

## Supplementary Material

vbaf332_Supplementary_Data

## Data Availability

The data underlying this article are available in the article and in its online supplementary material.

## References

[vbaf332-B1] Burgess DJ. Spatial transcriptomics coming of age. Nat Rev Genet 2019;20:317. 30980030 10.1038/s41576-019-0129-z

[vbaf332-B2] Chen T , Liu YX, Huang LJ. ImageGP: An easy‐to‐use data visualization web server for scientific researchers. Imeta 2022;1:e5. 38867732 10.1002/imt2.5 PMC10989750

[vbaf332-B3] Goodwin S , McPherson JD, McCombie W. Coming of age: ten years of next-generation sequencing technologies. Nat Rev Genet 2016;17:333–51. 27184599 10.1038/nrg.2016.49 PMC10373632

[vbaf332-B4] Li J , Miao B, Wang S et al; Hiplot Consortium. Hiplot: a comprehensive and easy-to-use web service for boosting publication-ready biomedical data visualization. Brief Bioinform 2022;23:bbac261. 10.1093/bib/bbac261 35788820

[vbaf332-B5] Lyu F , Han F, Ge C et al OmicStudio: a composable bioinformatics cloud platform with real-time feedback that can generate high-quality graphs for publication. Imeta 2023;2:e85. 38868333 10.1002/imt2.85 PMC10989813

[vbaf332-B6] Mullan KA et al ggVolcanoR: a Shiny app for customizable visualization of differential expression datasets. Comput Struct Biotechnol J 2021;19:5735–40. 34745458 10.1016/j.csbj.2021.10.020 PMC8551465

[vbaf332-B7] Wang H , Fu T, Du Y et al Scientific discovery in the age of artificial intelligence. Nature 2023;620:47–60. 37532811 10.1038/s41586-023-06221-2

[vbaf332-B8] Wickham H. ggplot2: Elegant Graphics for Data Analysis. New York: Springer-Verlag, 2016.

[vbaf332-B9] Zhao T , Wang Z. GraphBio: a shiny web app to easily perform popular visualization analysis for omics data. Front Genet 2022;13:957317. 36159985 10.3389/fgene.2022.957317 PMC9490469

