# Supplementary material for: ggplotAgent: a self-debugging multi-modal agent for robust and reproducible scientific visualization
Source: Bioinform Adv. 2026 Jan 2;6(1):vbaf332. doi: 10.1093/bioadv/vbaf332 (PMC12802885; doi:10.1093/bioadv/vbaf332)
Supplement: vbaf332_Supplementary_Data [file vbaf332_supplementary_data.zip › Supplementary Methods.docx]

**Supplementary Materials**

**ggplotAgent: A Self-Debugging Multi-Modal Agent for Robust and Reproducible Scientific Visualization**

Zelin Wang^1,^*, Yuanyuan Yin^1^, Jien Wang^2^, Haiyan Yan^1^, Xuan Xie^1,3,^* and Yiqing Zheng^1,^*

¹Guangdong Provincial Key Laboratory of Cancer Pathogenesis and Precision Diagnosis and Treatment, Joint Big Data Laboratory, Department of Medical Oncology, Shenshan Medical Center, Memorial Hospital of Sun Yat-sen University, Shanwei, China.

^2^Medical Research Center, Sun Yat-sen Memorial Hospital, Sun Yat-sen University, Guangzhou, China.

^3^Department of Thoracic Surgery, Sun Yat-Sen Memorial Hospital, Sun Yat-Sen University, Guangzhou, China.

*Corresponding author.

E-mail: [databio@163.com](mailto:databio@163.com).

**Supplementary Methods**

**Data Validation Agent**
This agent serves as a critical component to prevent execution failures due to data-request mismatch. It is explicitly told to account for common bioinformatics abbreviations and synonyms to allow for flexible interpretation. The prompt provides concrete examples, instructing the LLM that requests for log2FoldChange can be satisfied by columns named log2FC, logFC, or FoldChange; requests for an adjusted p-value can be met by p.adj, FDR, or q-value; and requests for gene name can be met by gene, gene_symbol, or symbol. Meanwhile, the agent is instructed to assume that simple mathematical transformations are possible (e.g., calculating -log10(FDR) from an FDR column) and not to flag these as errors.

**Plan Generation Agent**
This agent constructs a blueprint for the final R script, organized into a mandatory two-part structure. The first part, the "Data Preprocessing Plan," details all necessary data manipulation steps using functions from the dplyr and tidyr packages, such as using filter() to select specific rows or mutate() to create new columns, for instance, a significance category based on p-value and fold-change thresholds. The second part, the "Visualization Plan," strictly specifies the creation of a plot using ggplot2, defining the exact geom_ to be used, the aesthetic mappings within aes(), any modifications to scales via scale_ functions, the application of a base theme like theme_bw() or theme_classic(), and the final labels using labs(). A critical, hard-coded rule dictates that if text labels are requested for the plot, the plan must use ggrepel::geom_text_repel to ensure intelligent, non-overlapping label placement, with the entire blueprint being constrained to functions from only the tidyverse and ggrepel packages.

**Code Debugging Agent**
The Code Debugging agent is a sophisticated component whose prompt is dynamically constructed based on the type of error encountered. For a script execution failure, it receives the original plan, a data preview, the faulty script, and the captured stderr from the R process. Alternatively, for a visual quality assurance (QA) failure, it is given the same context but with QA feedback from an Image Checking Agent instead of a technical error message. The agent is strictly instructed to first articulate its reasoning and debugging strategy within an <thinking> block to promote meta-cognition and improve accuracy, after which it produces the corrected code. It also features a crucial escape hatch: if the agent determines the visual QA feedback is incorrect and the script is already correct, it must respond with the exact phrase NO_CHANGE_NEEDED, a mechanism designed to prevent erroneous QA feedback caused by limitations in the visual model.

**The Iterative Self-Debugging Loop**

The self-debugging loop is the core of ggplotAgent's robustness and is triggered by one of two conditions:

1. **Execution Failure:** The Code Execution Agent runs the R script. If it​ fails, the process proceeds​ to the Code Debugging Agent.
2. **Visual QA Failure:** If the script executes successfully, the graph proceeds to the Image Checking Agent. This agent analyzes the generated plot. If its evaluation is negative, it also directs the process to the Code Debugging Agent.

Once in the Code Debugging Agent, the agent uses the full context (plan, data, previous code, and the specific error or QA feedback) to generate a revised R script, routing​ it back to the Code Execution Agent and restarting the cycle. This loop continues until the plot passes visual QA or a maximum retry limit (defaulting to 3) is reached, at which point the process terminates and reports a user-facing error. Crucially, our design incorporates a persistent error history, enabling the system to learn from successive failures and systematically converge on a solution, rather than engaging in naive, repetitive attempts.

**Benchmark Construction**

We constructed a benchmark of 20 ggplot2 visualization tasks to evaluate model performance. The tasks were designed to cover a range of complexities. We evaluated the models across four distinct metrics, defined as follows:

1. **Code Executability Rate:** The percentage of generated code snippets that execute without raising any errors.
2. **Average Success Score:** A score on a 0-2 scale measuring how well the output visualization matches the user's prompt.
   - 0: Fails to address the core request.
   - 1: Partially addresses the request but has significant errors or omissions.
   - 2: Correctly and completely fulfills all explicit parts of the user's prompt.
3. **Average** **Insight Score:** A score on a -1 to +1 scale assessing the model's ability to introduce valuable, unprompted enhancements.
   - -1: The model makes an unprompted change that is detrimental to the plot's clarity or correctness.
   - 0: The model makes no unprompted changes or makes a neutral change.
   - +1: The model introduces a valuable enhancement (e.g., an appropriate scale transformation) not explicitly requested by the user that improves data interpretation.
4. **Average Publication-Ready Score:** A score on a 0-2 scale evaluating the aesthetic quality and professional polish of the resulting visualization.
   - 0: The plot is uninterpretable or visually chaotic.
   - 1: The plot is functional but requires significant manual adjustments to labels, colors, or layout to be considered professional.
   - 2: The plot is well-composed, with clear labels, an appropriate theme, and a professional aesthetic suitable for direct use in a publication.

**Pseudocode for ggplotAgent**

**
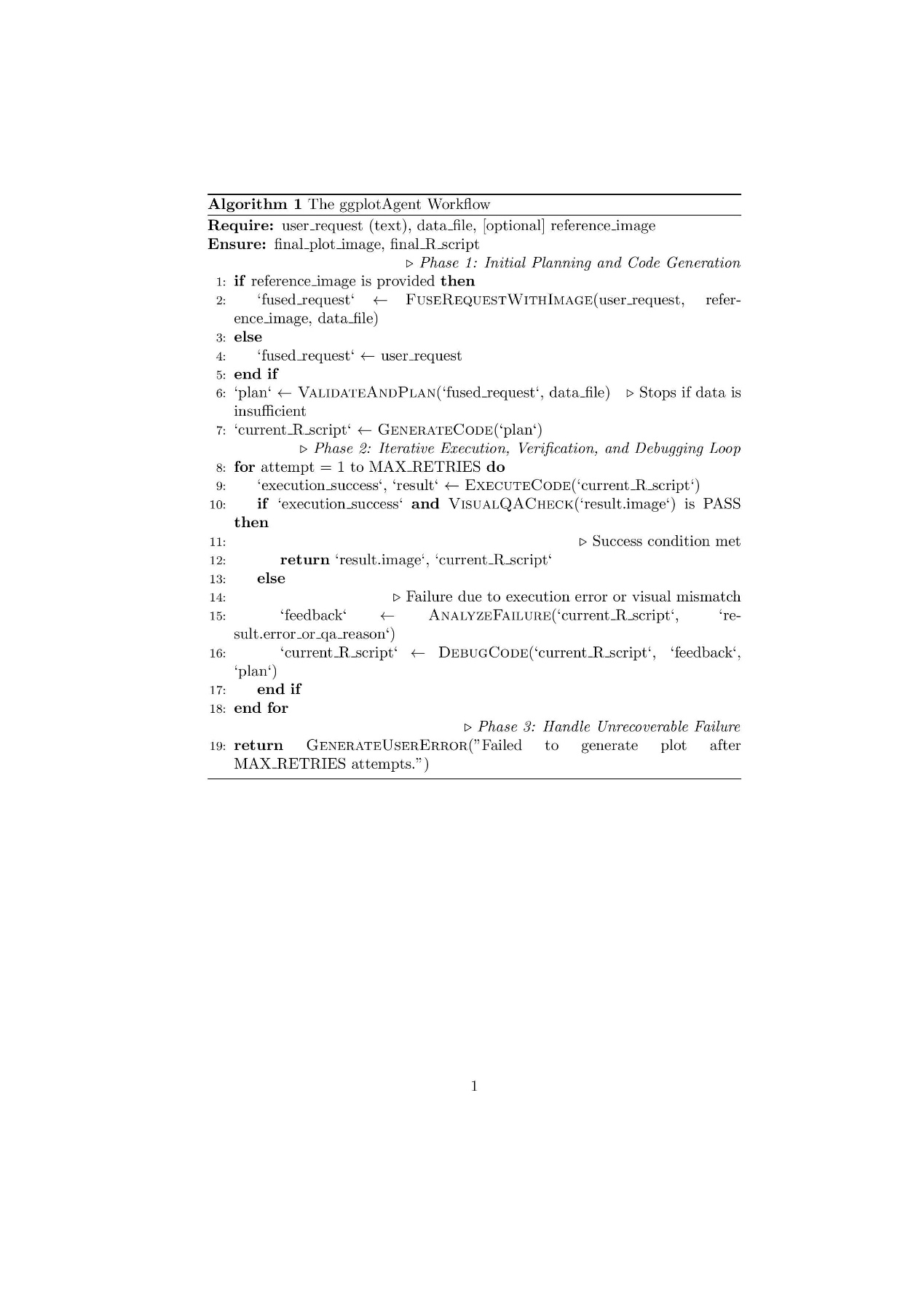
**

Algorithm 1 outlines the three primary phases of the ggplotAgent workflow.

1. Phase 1: Initial Planning and Code Generation (Lines 1-7): The process begins by optionally fusing the user's text prompt with a reference image to create a detailed specification (FuseRequestWithImage). The agent then validates the available data against this request and generates a comprehensive plotting plan (ValidateAndPlan). Based on this plan, an initial R script is generated (GenerateCode).
2. Phase 2: Self-Debugging Loop (Lines 8-18): This iterative loop is the core of the agent's intelligence. In each attempt, the current R script is executed (ExecuteCode). The outcome is subjected to a dual-verification process: checking for runtime errors and performing a visual quality assurance check on the generated image (VisualQACheck). A successful outcome requires passing both checks. If any check fails, the agent analyzes the error or visual feedback (AnalyzeFailure) and invokes a specialized debugging procedure (DebugCode) to generate a revised script for the next iteration.
3. Phase 3: Failure Handling (Line 19): If the agent cannot produce a satisfactory plot within the maximum number of retries, it terminates the process and returns a user-friendly error message, preventing infinite loops.

This structured, iterative approach allows ggplotAgent to move beyond simple code generation and actively refine its output, addressing both logical errors and subtle visual imperfections.
