# Supplementary figures and images for: ggplotAgent: a self-debugging multi-modal agent for robust and reproducible scientific visualization
Source: Bioinform Adv. 2026 Jan 2;6(1):vbaf332. doi: 10.1093/bioadv/vbaf332 (PMC12802885; doi:10.1093/bioadv/vbaf332)

A

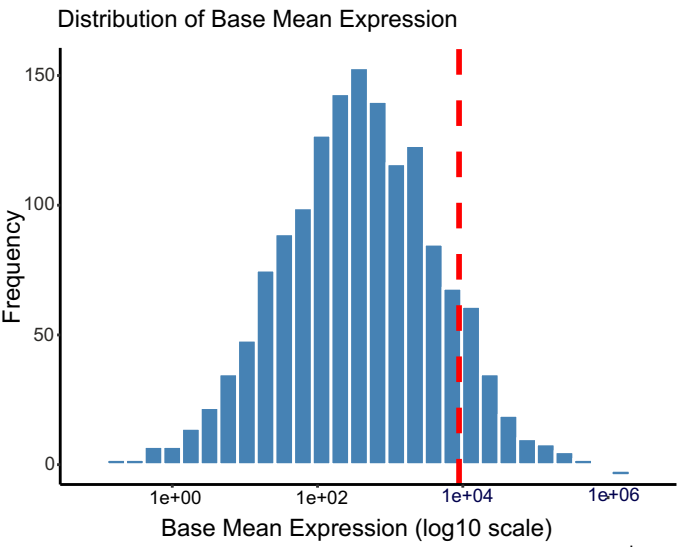

B

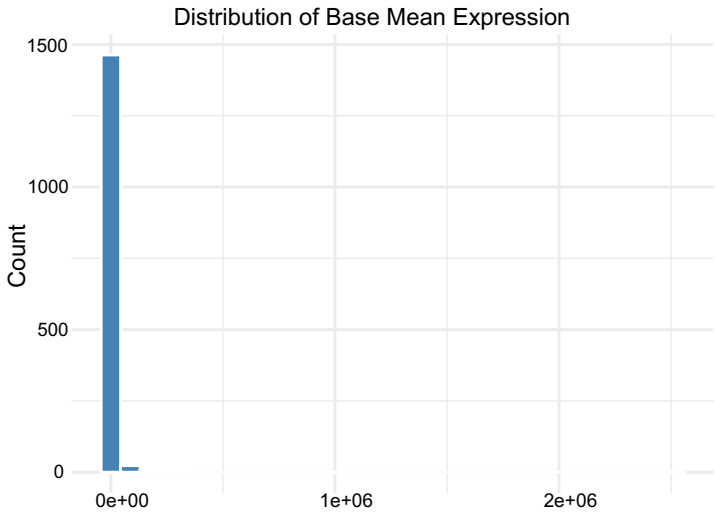

C

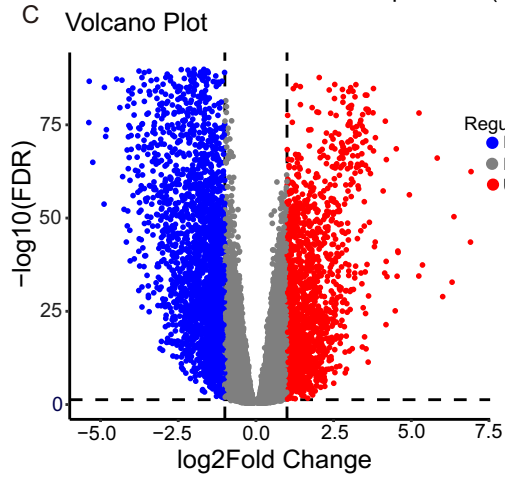

D

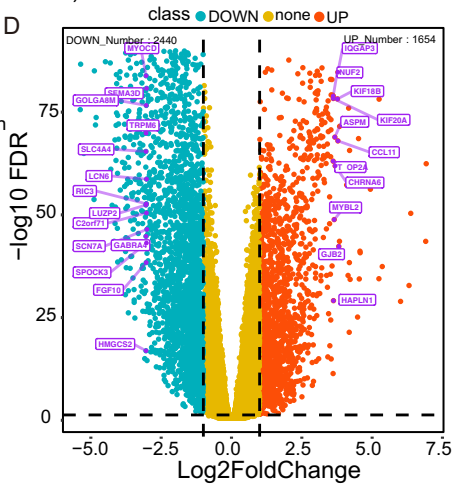

E

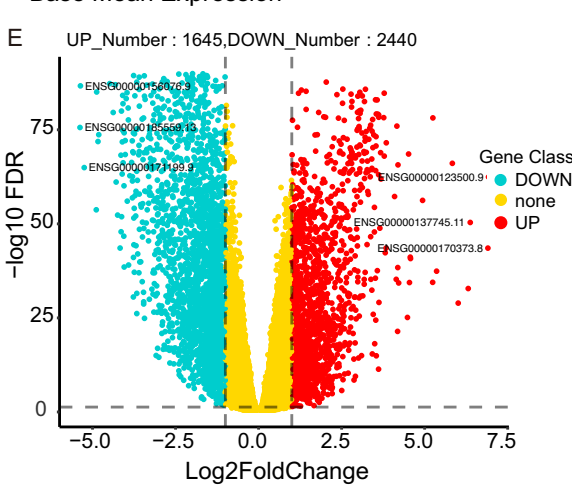

Supplement: vbaf332_Supplementary_Data [file vbaf332_supplementary_data.zip › Supplementary Fig 1.pdf]
